# Ten-year follow-up of thyroid epidemiology in Slovenia after increase in salt iodization

**DOI:** 10.3325/cmj.2011.52.615

**Published:** 2011-10

**Authors:** Katja Zaletel, Simona Gaberšček, Edvard Pirnat, Blaž Krhin, Sergej Hojker

**Affiliations:** Department of Nuclear Medicine, University Medical Centre Ljubljana, Ljubljana, Slovenia

## Abstract

**Aim:**

To assess iodine supply and follow thyroid epidemiology for ten years after an iodine increase from 10 to 25 mg of potassium iodide per kilogram of salt in 1999.

**Methods:**

In 2002 and 2003, we determined the thyroid size by palpation and ultrasound and measured urinary iodine concentration (UIC) in 676 schoolchildren from 34 schools throughout Slovenia. From 1999 to 2009, we followed the incidence of diffuse and nodular goiter, thyroid autonomy, Graves’ disease, and Hashimoto’s thyroiditis among adults in the stable catchment area of the University Medical Centre Ljubljana with 1 000 000 inhabitants.

**Results:**

In children, only 1% had a goiter grade 2 (visible and palpable thyroid gland), median thyroid volume was 5.8 mL, and median UIC was 148 µg/L. In adults, the incidence of diffuse goiter and thyroid autonomy decreased significantly (2009 vs 1999, rate ratio [RR], 0.16; 95% confidence interval [CI], 0.12-0.21 and RR, 0.73; 95% CI, 0.62-0.86, respectively), with a lower incidence in younger participants in 2009 (*P* < 0.001). The incidence of multinodular goiter and solitary nodule increased (2009 vs 1999, RR, 1.55; 95% CI, 1.35-1.79 and RR, 1.72; 95% CI, 1.49-1.99, respectively). No long-term changes were observed for Graves’ disease (2009 vs 1999, RR, 0.95; 95% CI, 0.81-1.13), while the incidence of Hashimoto’s thyroiditis increased strongly (2009 vs 1999, RR, 1.86; 95% CI, 1.64-2.12).

**Conclusions:**

The change from mildly deficient to sufficient iodine supply was associated with a marked change in the incidence of thyroid epidemiology – a significant decline in the incidence of diffuse goiter and thyroid autonomy and a marked increase in the incidence of Hashimoto’s thyroiditis.

Iodine is an essential element of thyroid hormones, crucial for the prevention of iodine deficiency disorders. Iodine supplementation is an important public health measure that influences the thyroid volume and determines the epidemiology of thyroid disorders. According to the World Health Organization (WHO) criteria, the main indicators of iodine supply are median urinary iodine concentration (UIC) and thyroid size in schoolchildren (1).

There are limited and sometimes conflicting longitudinal studies on epidemiology of thyroid disorders after the increase in iodine intake in areas with previously mild iodine deficiency. A few studies reported an early increase in the incidence of hyperthyroidism due to Graves’ disease and thyroid autonomy (2-4), in some cases followed by a decrease (2,5). Some reported an increased incidence of hypothyroidism (5), probably due to a higher incidence of thyroid autoimmunity (6-8). All investigators observed a decrease in the incidence of diffuse goiter in young population (3,9-11), while the data in the elderly are controversial (3,12).

The prevalence of goiter in Slovenia between the two world wars was up to 80% (13). In 1953, iodine prophylaxis was introduced with addition of 10 mg potassium iodide per kilogram of kitchen salt. In 1991 to 1994, an epidemiological study revealed a goiter grade 2 by WHO criteria in 11% of 1740 schoolchildren aged 13 years (14). The mean thyroid volume measured by ultrasonography was 7.2 mL and UIC was lower than 100 μg/g of creatinine in 73.7% of children, with a median of 82.9 μg/g of creatinine (14), which classified Slovenia a mildly iodine-deficient according to the WHO criteria. Therefore, in January 1999 the Ministry of Health issued a recommendation for mandatory salt iodization with 25 mg (within range of 20-30 mg) of potassium iodide.

In this study, we investigated the iodine supply and incidence of different thyroid disorders in Slovenia after the increase in salt iodization in 1999. We assessed the iodine supply in a population of schoolchildren using UIC and measurement of thyroid volume. We also performed a ten-year follow-up on the incidence of different thyroid disorders, including goiter, thyroid autonomy, Graves’ disease, and Hashimoto’s thyroiditis in the adult population.

## Participants and methods

### Schoolchildren study

In the epidemiological study conducted in 2002 and 2003, we included 676 schoolchildren aged 13 years, 343 girls and 333 boys, from the same 34 public elementary schools as in the study conducted in 1991 to 1994 (14). At that time, schools were selected from all regions throughout Slovenia considering the number of 13-year olds in each region. In every school, one class of schoolchildren of appropriate age was included. We determined the size of their thyroid gland by inspection and palpation, separately by two independent expert examiners according to WHO criteria: grade 0 (not visible, not palpable thyroid gland), grade 1A (not visible, palpable thyroid gland), 1B (visible at the extended neck position, palpable thyroid gland), 2 (visible at the normal neck position, palpable thyroid gland). Thyroid volume was measured using an Aloka SSD-900 ultrasound with 5 MHz probe and Brunn’s formula (15). UIC was measured using commercially available kit (Urinary iodine assay kit, Bioclone Australia Pty Limited, Sydney, Australia), based on the improved Sandell-Kolthoff reaction, and samples were digested with ammonium persulphate on microtiter plate by specially designed sealing cassette. UIC was expressed in microgram per liter. The study was approved by the National Medical Ethics Committee and the participants signed a written informed consent prior to enrolment in the study.

### Identification of new cases of thyroid disorders in adult Slovenian population

We consecutively examined adult patients referred for the first time to the Thyroid Department at the University Medical Centre Ljubljana. It is the only institution with thyroid specialists and with facilities for thyroid scintigraphy in the area, covering a half of Slovenia. This catchment area with approximately 1 million inhabitants has remained unchanged for more than 20 years. Patients were referred by general practitioners under the suspicion of thyroid dysfunction or goiter. Each patient was examined by one out of six thyroid specialists working at this department. We continuously identified all new cases of different thyroid disorders. The data in the period from January 1, 1999 to December 31, 2009 were obtained from the computer-based register of the Department of Nuclear Medicine.

### Diagnostics of thyroid disorders

All patients were clinically examined. We measured thyrotropin (TSH), free thyroxine (T_4_), free triiodothyronine (T_3_), thyroid peroxidase antibodies (TPOAb), and thyroglobulin antibodies (TgAb), and performed thyroid ultrasound. Diffuse and nodular goiter was diagnosed on the basis of ultrasound picture, while thyroid function was normal and TPOAb and TgAb were negative. Patients with nodular goiter presented either with multinodular goiter or with solitary nodule. Thyroid autonomy was diagnosed by biochemical hyperthyroidism and thyroid scintigraphy with 99m-pertechnetate and patients were negative for TSH receptor antibodies (TSHRAb). Patients with Graves’ disease were hyperthyroid and positive for TSHRAb, while patients with Hashimoto’s thyroiditis presented with different thyroid function and elevated TPOAb and/or TgAb. In both, ultrasound showed characteristic hypoechoic picture. Hypothyroid Hashimoto’s thyroiditis patients presented with elevated TSH and normal or decreased free thyroid hormones.

### Statistics

Statistical analysis was performed with the Statistica software (StatSoft, Tulsa, OK, USA). Goiter volume and UIC in schoolchildren were expressed with median values. The correlation between thyroid size estimated by palpation and thyroid volume was calculated using Spearman correlation test.

The incidence rates were evaluated using rate ratios (RR) with baseline value in 1999 and 95% confidence intervals (CI), calculated after log transformation of the respective rates. Calculations were made assuming the Poisson distribution of cases (*http://www.quantitativeskills.com/sisa/statistics*). We compared the age of patients with different thyroid diseases in the year of the increase in iodine intake (year 1999) and ten years later (year 2009). The selection of these two years was based on our assumption and on previous observations (2,16) that more than five years was necessary for the stabilization of the epidemiological situation after such an increase in iodine supply. Ages were expressed as medians and range. Groups were compared using Mann-Whitney *U* test. *P* value below 0.05 was considered significant.

## Results

**Figure 1:**
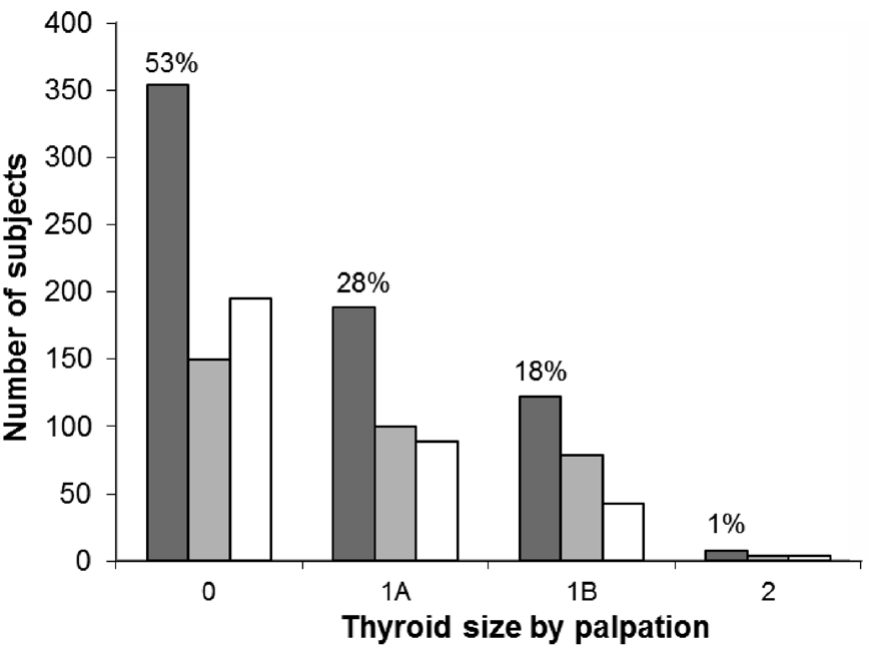
Thyroid size in 676 schoolchildren after the increase in iodine intake in Slovenia estimated by inspection and palpation. Grade 0 (not visible, not palpable thyroid gland), grade 1A (not visible, palpable thyroid gland), 1B (visible at the extended neck position, palpable thyroid gland), 2 (visible at the normal neck position, palpable thyroid gland). Closed bars – all children, gray bars – boys, open bars – girls.

**Figure 2:**
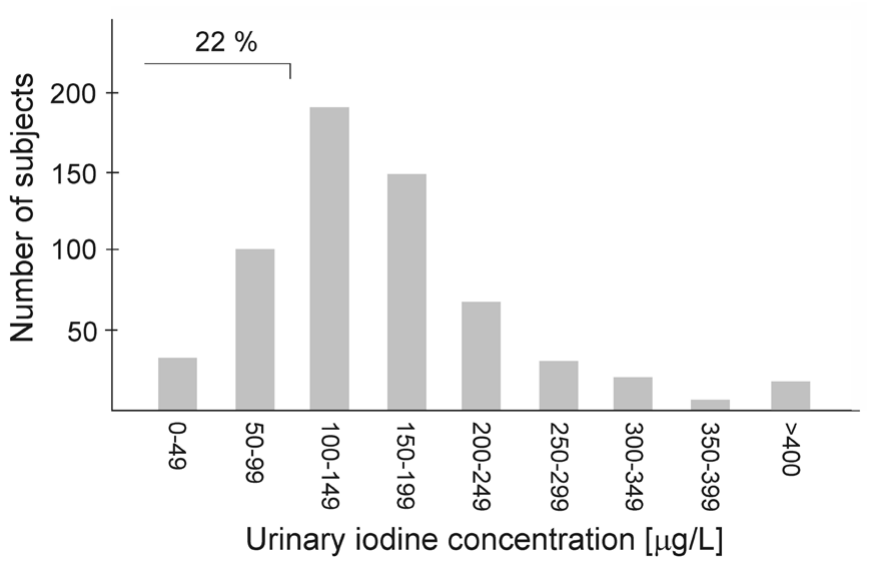
Urinary iodine concentration in 676 schoolchildren after the increase in iodine intake in Slovenia. As indicated in the figure, 22% of schoolchildren had urinary iodine concentration below 100 μg/L.

There were 53% of children with thyroid gland grade 0, 28% with 1A, 18% with 1B, and only 1% with grade 2 (Figure 1). Median thyroid volume measured by ultrasound was 5.8 mL (range, 1.9-30.3 mL); in girls 5.9 mL (range, 2.2-24.2 mL) and in boys 5.7 mL (1.9-30.3 mL). Median UIC was 148 µg/L (range, 2-1744 µg/L); in girls 148 µg/L (range, 6-1744 µg/L) and in boys 148 µg/L (range, 2-741 µg/L). Median UIC was below 100 µg/L in only 22% of children (Figure 2). The correlation between the results of palpation and thyroid volume was significant (r = 0.46, *P* < 0.001).

**Figure 3:**
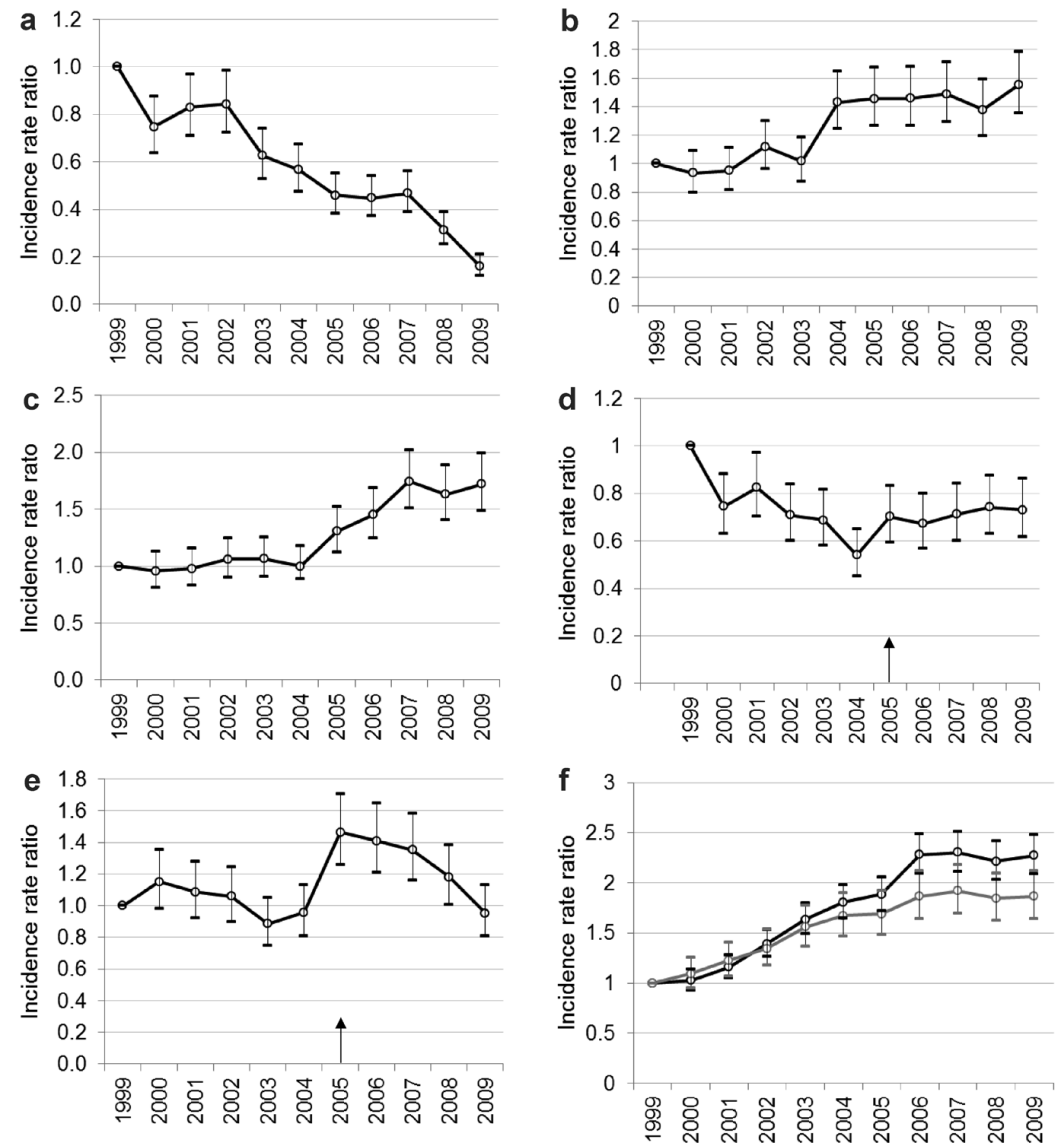
The rate ratio of thyroid diseases incidence in the period from 2000 to 2009 vs baseline in 1999, the year when the increased salt iodization was introduced. 95% confidence intervals of the rate are indicated by vertical bars. The data refer to diffuse goiter (**A**), multinodular goiter (**B**), solitary nodule (**C**), thyroid autonomy (**D**), Graves’ disease (**E**), and Hashimoto’s thyroiditis (**F**) where hypothyroid patients are shown with gray line. In Figures **E** and **D**, the pointer indicates the introduction of a new (second) generation of the thyrotropin receptor antibody assay. Arrows indicate introduction of new methods.

After the increase in iodine intake, the incidence of diffuse goiter in adult Slovenian population gradually decreased from 35.3/100 000 in 1999 to more than an 80% lower value in 2009 (vs baseline, RR, 0.16; 95% CI, 0.12-0.21) (Figure 3A). Patients with diffuse goiter were significantly older in 2009 than in 1999 (Table 1). The incidence of multinodular goiter and solitary nodules was 32.7/100 000 and 28.6/100 000, respectively, in 1999 but during the last five years until 2009 it slowly increased to 55% and 72% higher levels, respectively (RR, 1.55; 95% CI, 1.35-1.79 and RR, 1.72; 95% CI, 1.49-1.99, respectively) (Figure 3B and 3C). In both diseases, there was no significant difference in the age of patients before and after the iodine increase (Table 1).

**Table 1:** Age of patients with different thyroid diseases after the increase in salt iodization in Slovenia in 1999 from 10 to 25 mg potassium iodide per kilogram of salt

	1999		2009		
Thyroid disease	number	median age in years (range)	number	median age in years (range)	P*
Diffuse goiter	353	38 (14-84)	56	53 (16-88)	<0.001
Multinodular goiter	327	59 (18-92)	508	60 (15-93)	0.058
Solitary nodule	286	48 (15-90)	492	50 (15-89)	0.861
Thyroid autonomy	327	69 (19-91)	239	72 (27-92)	<0.001
Graves’ disease	278	41 (15-78)	265	44 (16-82)	0.101
Hashimoto’s thyroiditis (HT), total	732	51 (16-89)	1664	50 (15-97)	0.211
Non-hypothyroid HT^†^	363	48 (16-88)	976	48 (15-88)	0.629
Hypothyroid HT^†^	369	54 (18-89)	688	54 (15-97)	0.797

*Mann-Whitney U test.

†*P* < 0.001 when comparing age of non-hypothyroid and hypothyroid HT patients.

The baseline incidence of thyroid autonomy was 32.7/100 000 and by 2009 it gradually and significantly decreased to a 27% lower value (RR, 0.73; 95% CI, 0.62-0.86) (Figure 3D). Patients with thyroid autonomy were significantly older than other thyroid patients (*P* < 0.001). Interestingly, in 2009, the median age of newly diagnosed thyroid autonomy patients was significantly higher than in 1999 (Table 1).

The baseline incidence of Graves’ disease was 27.8/100 000 and it did not change significantly until 2005, when we introduced a more sensitive second generation TSH receptor antibody assay. However, in 2009 the incidence decreased to the baseline level (vs baseline, RR, 0.95; 95% CI, 0.81-1.13) (Figure 3E). The age of patients with Graves’ disease did not change significantly (Table 1).

The baseline incidence of Hashimoto’s thyroiditis was 73.2/100 000 and it gradually increased during the first eight years to the levels more than twice as high as before. Since 2006, the incidence remained stable, with a rate of 166.4/100 000 in 2009 (vs baseline, RR, 2.27; 95% CI, 2.08-2.48). The observation was similar in patients with hypothyroid Hashimoto’s thyroiditis, the incidence of which was 36.9/100 000 in 1999 and 68.8/100 000 in 2009 (vs baseline, RR, 1.86; 95% CI, 1.64-2.12) (Figure 3F). The age of patients with Hashimoto’s thyroiditis was similar in 2009 and 1999, and in both periods hypothyroid patients were significantly older at the disease presentation than patients with euthyroid Hashimoto’s thyroiditis (*P* < 0.001) (Table 1). In spite of a marked increase in Hashimoto’s thyroiditis incidence, the proportion of patients with hypothyroid Hashimoto’s thyroiditis was significantly lower than the proportion of those with non-hypothyroid Hashimoto’s thyroiditis in 2009 than in 1999 (χ^2^ = 16.57, *P* < 0.001).

## Discussion

The present study confirmed that after the increase in salt iodization in Slovenia there was an adequate iodine supply, with a median UIC in schoolchildren of 148 µg/L and a prevalence of goiter below 1%. The ten-year follow-up showed a marked change in thyroid epidemiology in adults, with a gradual decrease in the incidence of diffuse goiter and thyroid autonomy, an increase in the incidence of Hashimoto’s thyroiditis and nodular goiter, but only a transient increase in the incidence of Graves’ disease. Although the incidence was estimated on the basis of new cases registered in thyroid department, which might be considered a limitation, we believe that a very large and stable catchment area enabled us an excellent approximation of the real incidence.

This study showed that according to WHO criteria Slovenia was an iodine-sufficient area in terms of UIC, thyroid size, and goiter prevalence in schoolchildren (1). We consider median UIC to be adequate, although we were not able to make a precise comparison with our previous study (14), conducted from 1991 to 1994, since the measure of UIC in that study was iodine per gram of creatinine, while in this study it was iodine per liter of urine. The experience in the last decade showed that the latter was a better measure of iodine supply in schoolchildren (17). We were able to compare the thyroid volume, which significantly decreased from 7.2 mL to 5.8 mL (14). Other reports on schoolchildren following improved iodine supply have observed a decreased goiter prevalence (10,11,18,19).

The reports on goiter in adults are limited and conflicting. Our data showed a significantly lower incidence of diffuse goiter after improved iodization, which is in accordance with a Danish observational study (12), as well as with two interventional studies that showed a significant decrease in thyroid volume in patients with euthyroid diffuse goiter (20,21). In a small group of Swiss patients, no change in thyroid volume before and after the increase in iodine supplementation has been observed (22). In iodine-sufficient Austrian population, a small cross-sectional study revealed a higher prevalence of both diffuse and multinodular goiter in the age group older than 40 years (3). Similar is our observation that patients diagnosed with diffuse goiter were significantly older ten years after increased iodization, which indicates that there is a decrease in the incidence of diffuse goiter in the younger population. We found an increasing incidence of nodular goiter after increased iodization, as opposed to a study on Danish population (12). We have no scientific explanation for such a finding but we believe that this phenomenon probably is not associated with iodine supplementation but is a reflection of more neck ultrasounds performed in Slovenia in the last five years. However, despite more frequently diagnosed nodular goiter, there was a decrease in the incidence of highly malignant thyroid carcinoma (23).

The data on the incidence of thyroid autonomy after increased iodization are scarce. While the reports from Switzerland and Austria indicate a short-term increase (2,16,24), we observed a significant decrease already in the first year after increased iodization. Such finding is in line with investigations carried out in Switzerland (2,16). Both our and Austrian data show that after the increase in iodine intake, there were more thyroid autonomy patients belonging to an older age-group. One might assume that a long-term sufficient iodine intake reduces the occurrence of goiter and thyroid autonomy especially in younger population, which might explain the older age of thyroid autonomy patients in 2009.

The incidence of Graves’ disease did not change considerably during the 10-year follow-up, which does not confirm the results of other studies. In Switzerland, the initial increase in incidence was followed by a decrease to a level lower than before the increased iodization (2,16). In Austrian population, the Graves’ disease incidence was still high 5 years after increased iodization (24). The differences in Graves’ disease incidence might be influenced by different observation periods and by differences in diagnostics caused by evolution of TSH receptor antibody assay.

A large epidemiological study has reported a high incidence of thyroid antibodies and hypothyroidism in area with adequate iodine intake (25). These data are in accordance with our study, as well as with several other studies in which the incidence of thyroid antibodies and the prevalence of hypothyroidism increased after the increase in iodization (3,5,19,26-28). The same age of affected participants before and after iodine increase indicates the importance of genetic background, while the disease is equally accelerated by iodine in all age groups.

In conclusion, in an iodine-sufficient area of Slovenia, the change from mildly deficient to sufficient iodine supply resulted in a long-term change in thyroid disease pattern. A significant decrease in diffuse goiter and thyroid autonomy, predominantly in younger population, indicates the protective role of adequate iodine intake in their pathogenesis. The increased incidence of Hashimoto’s thyroiditis in all age groups also confirms that increase in iodine intake strengthens thyroid autoimmunity regardless of age, probably in genetically predisposed individuals.
